# The Outcome of Surgical Treatment for the Neuropathic Diabetic Foot Lesions—A Single-Center Study

**DOI:** 10.3390/life12081156

**Published:** 2022-07-29

**Authors:** Florin Bobirca, Catalin Gabriel Smarandache, Anca Bobirca, Cristina Alexandru, Dan Dumitrescu, Anca Pantea Stoian, Cristina Bica, Lacramioara Aurelia Brinduse, Anca Musetescu, Daniela-Elena Gheoca-Mutu, Sebastian Isac, Ioan Ancuta

**Affiliations:** 1 Carol Davila University of Medicine and Pharmacy, 050474 Bucharest, Romania; florin.bobirca@umfcd.ro (F.B.); catalin.smarandache@umfcd.ro (C.G.S.); dan-andrei.dumitrescu@drd.umfcd.ro (D.D.); anca.stoian@umfcd.ro (A.P.S.); ioana-cristina.bica@drd.umfcd.ro (C.B.); lacramioara.brinduse@umfcd.ro (L.A.B.); daniela.mutu@drd.umfcd.ro (D.-E.G.-M.); sebastian.isac@umfcd.ro (S.I.); ioan.ancuta@umfcd.ro (I.A.); 2 Surgery Department, Dr. Ion Cantacuzino Clinical Hospital, 011437 Bucharest, Romania; 3 Department of Surgery, University Emergency Hospital, 050098 Bucharest, Romania; 4 Internal Medicine and Rheumatology Department, Dr Ion Cantacuzino Clinical Hospital, 011437 Bucharest, Romania; maria-cristina.steopoaie@rez.umfcd.ro; 5 Department of Diabetes, Nutrition and Metabolic Diseases, 050474 Bucharest, Romania; 6 Department of Public Health and Management, Carol Davila University of Medicine and Pharmacy, 050474 Bucharest, Romania; 7 Rheumatology Department, Craiova University of Medicine, 200349 Craiova, Romania; anca.musetescu@umfcv.ro; 8 Department of Plastic and Reconstructive Surgery, “Prof. Dr. Agrippa Ionescu” Clinical Emergency Hospital, 011356 Bucharest, Romania; 9 Department of Physiology and Neuroscience, Carol Davila University of Medicine and Pharmacy, 050474 Bucharest, Romania

**Keywords:** diabetic foot surgery, amputation, diabetes mellitus, gangrene

## Abstract

The prevalence of diabetic foot complications is continuously increasing as diabetes has become one of the most important “epidemics” of our time. The main objective of this study was to describe the appropriate surgical intervention for the complicated neuropathic diabetic foot; the secondary goal was to find the risk factors associated with minor/major amputation and good or adverse surgical outcomes. This is an observational, retrospective study conducted between 1 January 2018 and 31 December 2019, which included 251 patients from the General Surgery Department at the Dr I. Cantacuzino Clinical Hospital in Bucharest with type II diabetes mellitus and neuropathic diabetic foot complications. The surgical conditions identified at admission were the following: osteitis (38.6%), infected foot ulcer (27.5%), gangrene (20.7%), infected Charcot foot (3.6%), non-healing wound (3.6%), necrosis (3.2%), and granulated wound (2.8%). We found that a minor surgical procedure (transmetatarsal amputation of the toe and debridement) was performed in 85.8% of cases, and only 14.2% needed major amputations. Osteitis was mainly associated with minor surgery (*p* = 0.001), while the gangrene and the infected Charcot foot were predictable for major amputation, with OR = 2.230, 95% CI (1.024–4.857) and OR = 5.316, 95% CI (1.354–20.877), respectively. Admission anemia and diabetic nephropathy were predictive of a major therapeutical approach, with *p* = 0.011, OR = 2.975, 95% CI (1.244–8.116) and *p* = 0.001, OR = 3.565, 95% CI (1.623–7.832), respectively. All the major amputations had a good outcome, while only several minor surgeries were interpreted as the adverse outcome (*n* = 24). Osteitis (45.8%) and admission anemia (79.2%) were more frequently associated with adverse outcomes, with *p* = 0.447 and *p* = 0.054, respectively. The complicated neuropathic diabetic foot requires a surgical procedure mainly associated with a good outcome.

## 1. Introduction

The prevalence of the complications of diabetes mellitus is continuously increasing as it has become one of the most important “epidemics” of our time.

Major amputation is the most debilitating complication for a patient with diabetic foot syndrome. However, from the surgeon’s point of view, the risk for amputation can be seriously decreased by adapting surgery to the patient’s particularities.

In most cases, foot lesions appear after at least ten years of diabetes evolution of the skin, skeleton, muscles, vessels, and nerves. These changes define the notion of diabetic foot syndrome.

The pathology of the diabetic neuropathic foot (DNF) is the “positive component” of the complications of diabetic foot because the patient with a predominance of neuropathic complications has the highest chance of not reaching a major amputation [1,2]. This is due to the minimal vascular damage that has two benefits: on the one hand, it makes it possible for antibiotics to access the site of infection, and on the other hand, the vascular support facilitates the appearance of granulation tissue and wound healing.

The most common types of lesions specific to neuropathic complications of diabetic foot are ulcers, osteitis, neuropathic gangrene, Charcot foot, etc.

Risk factors for these types of neuropathic lesions are the period from the onset of diabetes, the patient’s age, compensation for metabolic disease, history of ulceration, and the presence and degree of retinopathy, especially polyneuropathy. It is now known that 50% of diabetic foot injuries occur based on the predominance of neuropathy [3,4].

Several pathogenetic mechanisms are taken into account: mainly malfunction of polyol and myo-inositol metabolism, reduction of Na/K-ATPase, endoneurial microvascular deficits with consecutive ischemia, formation of oxygen radicals, neurotrophic disorder (IGF-I, NGF), defective axonal transport, and non-enzymatic glycosylation of neuronal structural and transportation protein [5,6,7,8,9,10].

It is essential to differentiate between non-infected DNF lesions that require conservative treatment and septic lesions that need a surgical treatment well adapted to them.

### 1.1. The Clinical Diagnosis 

The clinical diagnosis of the diabetic neuropathic foot is partially established from the moment of inspection when a change in the foot’s shape can be observed due to the damage to the skeletal system. These changes in shape, depending on where they are located, define the following anatomical–clinical situations:

1. Charcot osteoarthropathy is the most typical lesion for diabetic peripheral neuropathy and occurs due to repeated, unnoticed microtraumas that lead to diffuse inflammation of the skeleton [11]. The collapse of the plantar arch is a consequence of this inflammation, with extensive changes in the foot biomechanics that will later require complex, multidisciplinary treatment: podiatry, surgery, orthopedics, and rheumatology [9,10].
2. Hammertoe, which is often associated with hallux valgus, is seen in people wearing shoes that cause a foot malposition.
3. Diabetic foot ulcerative lesions.
4. Toe gangrene.
5. Toe osteitis.

In addition to the anatomical–clinical forms described above, the typical skin lesions for peripheral neuropathy are plantar anhidrosis, callus, absence of hair, and onychomycosis. Pelvic limb venous dilatation due to the opening of the arteriovenous anastomosis is associated with the severity of neuropathy and often with Charcot’s foot [5].

### 1.2. Paraclinical Tests 

Paraclinical tests of the neuropathic diabetic foot include tests for nerve conduction velocity, as it decreases in patients with advanced neuropathy. Vibration sensitivity can be tested by using a 128 Hz tuning fork on the medial ankle, first toe, and fifth toe. The perception of tactile sensitivity is achieved with the help of the Semmes–Weinstein test. This practical test determines if the patient maintains pain sensitivity in case of plantar lesions. The test is performed on the plantar surface of the first, fourth, and fifth metatarsals. Foot radiography may indicate changes in the skeleton (such as osteolysis) or the soft tissue. MRI is used to diagnose Charcot foot changes [9,10].

### 1.3. Prophylaxis of Lesions/Infections

First of all, the diet should be considered, promoting the best possible control of diabetes, combined with physical activity that maintains adequate blood flow in the lower limbs [12,13]. In addition, recommended exercises are those that help develop joint mobility and reduce plantar pressure. Another important measure is custom footwear, used for pressure relief and adapted to the patient’s lesion [14,15].

Daily self-inspection of the feet by patients is a first step in screening for this disease.

### 1.4. The Treatment 

The treatment of diabetic foot is extremely complex and involves a multidisciplinary team: a podiatrist, diabetologist, surgeon, orthopedist, plastic surgeon, etc. The severity of diabetic foot lesions is obviously related to the degree of compensation of the underlying disease. Thus, first of all, a metabolic settlement, by adjusting the insulin doses or oral antidiabetics, represents the first step in the treatment algorithm of this pathology. In selected cases, such as Charcot’s foot, the orthopedist has an important role in this multidisciplinary team. The medical treatment of DNF involves the administration of antibiotics, vasodilators, and neurotrophins. The initial antibiotic therapy is broad-spectrum, and antibiotics will be administered according to the antibiogram 72 h after sampling. Often, diabetic patients have multigerm flora in their lesions, sometimes multidrug-resistant, so antibiotic treatment can be challenging [5,9,10].

Conservative treatment of DNF consists of applying local topics and is an accessible method in treating ulcerations caused by peripheral neuropathy in the early stages. There are also variants, such as special dressings impregnated with different substances. Hydrocolloids and hydrogels perfectly absorb exudate from the wound and its hydration to allow accelerated granulation. Another option is iodine dressings, which are useful in preventing germs from spreading in the wound [13,16,17,18,19].

Complementary but beneficial methods in the treatment of DNF are:

- Hyperbaric oxygen therapy is one of the most important therapeutic resources. Some studies confirm that after two weeks from the initiation of this therapy, the ulcers begin to heal and decrease in size, mentioning that complete healing remains a long process [20,21].
- Treatment with negative pressure on wounds and diabetic foot ulcers is an effective adjunctive therapy. From the mechanism of action, it is based on the creation of an anti-inflammatory and pro-angiogenic effect, with the stimulation of the growth factors that will lead to the appearance of the granulation tissue [22,23,24].
- Plastic surgery techniques cover defects in the foot’s soft tissue.

### 1.5. DNF Surgical Treatment

Debridement is essential in the surgical treatment of neuropathic ulcers and diabetic foot wounds. This has the role of reaching healthy tissue, and the indications are infected neuropathic ulceration, advanced osteitis, gangrene, abscess, etc.

Transmetatarsal toe amputation is indicated for toe gangrene or neuropathic ulcer associated with infection and bone destruction [25,26,27,28].

Below-knee amputation is indicated in the case of extensive gangrene of the foot, in which tissue destruction is an important and advanced septic syndrome [29,30,31,32].

The next level of major amputation in the above-knee amputation, which is the most disabling intervention. It is indicated in borderline cases for DNF lesions, especially in extensive gangrene, with spindles and tissue destruction of up to one-third of the upper leg [33,34,35,36].

### 1.6. Objectives

The main objective of this study is to describe the surgical outcome for the neuropathic diabetic foot in our tertiary center specialized in diabetic foot treatment. As secondary objectives, we aimed to find the risk factors associated with minor/major amputation and with good (defined by the proper healing of the wound) or adverse surgical outcomes (as post-surgical complications such as infection, the necessity for reintervention).

## 2. Material and Methods

This is a retrospective, observational study conducted between 1 January 2018 and 31 December 2019, which included 251 patients from the General Surgery Department at the Dr I. Cantacuzino Clinical Hospital in Bucharest.

The inclusion criteria were patients aged over 18 years with the diagnosis of type II diabetes complicated by diabetic neuropathy, with lesions of the diabetic neuropathic foot, who underwent minor (toe/transmetatarsal resection, debridement) or major (below-knee amputation, above-knee amputation) surgery.

The diagnosis of diabetic neuropathic foot was established by using neuropathy tests (tactile, thermal, pain and vibratory senses) and X-rays (especially for osteolysis).

Exclusion criteria were patients with type I diabetes, predominantly arteriopathy lesions, patients without diabetes, and patients who did not require surgery.

Patient demographics are based on observation sheets registered in the hospital database, with patients agreeing in writing to have their data collected for scientific purposes in accordance with the Declaration of Helsinki.

### Statistical Analysis

Excel and SPSS v19 (Statistical Package for the Social Sciences Inc., Chicago, IL, USA) were used for data processing. Categorical variables are presented as absolute numbers and respective percentages. Continuous variables are reported as median and standard deviation. To determine any significant associations, the data were reported using an odds ratio (OR) with 95% confidence interval (CI) and *p* values. A significant result was considered for all tests at a *p*-value < 0.05.

## 3. Results

Characteristics of the studied cohort are shown in Table 1. Males were predominant, accounting for 70.9% (178 individuals). The average age of patients enrolled at the time of admission was 61.1 years, with 63.7% (163) being in the age group older than 50 years and less than or equal to 70 years. A total of 148 patients (59.0%) had urban residence and 103 (41.0%) lived in rural areas. The duration from the time of diagnosis of diabetes to the time of surgery was analyzed, with a mean of 11.56 ± 6.5 years, and 45.0% (113) of the patients were insulin-dependent. The mean glycemia at admission was 200.83 ± 102.0 mg/dL (Table 1).

Regarding the complications of diabetes, 19.9% of patients also had diabetic retinopathy, and 17.1% were diagnosed with diabetic nephropathy. In addition, over half of the study group, 63.3%, suffered from cardiovascular disease at the time of surgery (cardiovascular disease is defined by the presence of at least one of the following: coronary heart disease, stroke, high blood pressure) (Table 1).

At admission, the anemic status was analyzed, and the results show that 61% had hemoglobin values ≤ 12 g/dL for women and ≤13 g/dL for men. In addition, leukocytosis with a defined leukocyte value greater than 11,000/mL at the time of admission was present in 35.5% of patients (Table 1).

It was revealed that osteitis and ulcer were the most routine diagnoses of our patients with predominant neuropathic foot lesions. Therefore, an extensive analysis was performed to identify the characteristics of these two subgroups, osteitis and ulcer, and a comparison of these subgroups and the rest of the cohort is shown in Table 1.

Osteitis was the main surgical diagnosis identified (97 cases out of 251), and it was demonstrated that male sex and diabetes duration were often associated with this entity, with *p* = 0.012, OR = 0.496. 95% CI (0.284–0.861) and *p* = 0.015, OR = 0.949, 95%CI (0.909–0.991), respectively.

At admission, several blood laboratory tests were evaluated. The results showed that patients having higher hyperglycemia, anemia, and leukocytosis were more likely to have one of the other diagnoses registered in the study besides osteitis, with a significant *p*-value (*p* = 0.007, OR = 0.997 95%CI (0.994–0.999); *p* = 0.001, OR = 0.426 95% CI (0.252–0.719); *p* = 0.023, OR = 0.529 95% CI (0.304–0.919), respectively).

**Figure 1:**
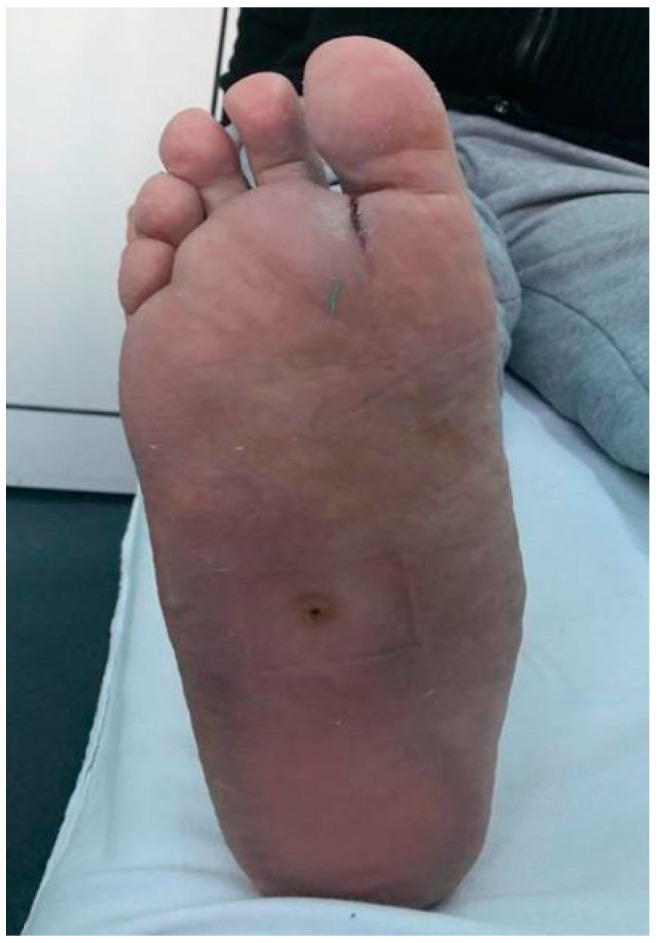
Neuropathic diabetic foot ulceration associated with Charcot Osteoarthropathy. Dr. I. Cantacuzino Clinical Hospital Collection.

The same statistics were applied to evaluate the “ulcer” subgroup, but neither of the characteristics were strongly associated, with all the *p* values being higher, *p* > 0.005. Regarding the surgical approach, the majority (88.40%) of the patients with foot ulcers needed minor surgery (Figure 1).

**Figure 2:**
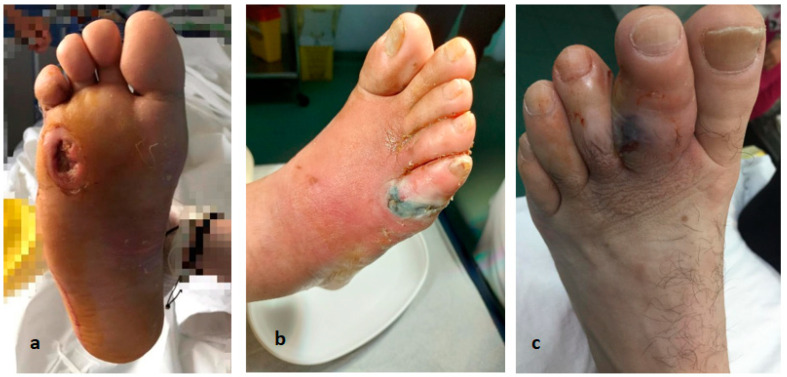
(**a**) Plantar ulcer (**b**) Toe gangrene (**c**) Toe osteitis. Dr. I. Cantacuzino Clinical Hospital Collection.

The surgical conditions identified at admission were the following: osteitis (38.6%), infected foot ulcer (27.5%), gangrene (20.7%), infected Charcot foot (3.6%), non-healing wound (3.6%), necrosis (3.2%), granulated wound (2.8%) (Table 2) (Figure 2).

**Figure 3:**
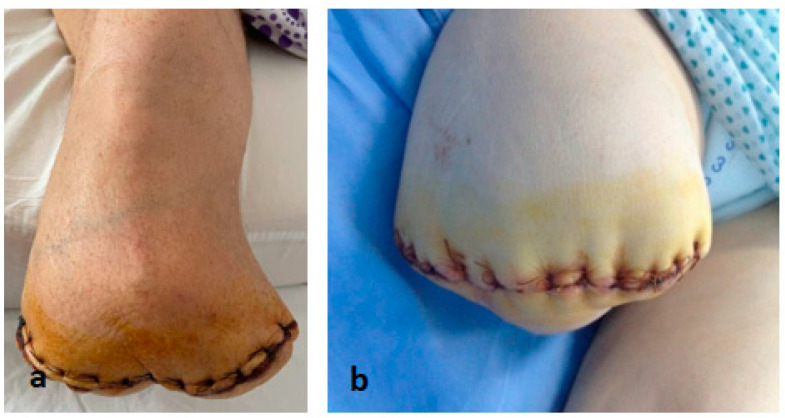
(**a**) Below-knee amputation (**b**) Above-knee amputation.

Regarding the type of surgery to which the patients were subjected, in most situations (211 cases), a minor operation was performed, such as transmetatarsal amputation of the toe (143 cases, 57%), and debridement was performed in 68 cases (27.1%). Major interventions (amputations) were performed in 35 patients (7.6% below-knee and 6.6% above-knee) (Figure 3). Five patients with skin grafts and secondary sutures were not included in the following comparison because they were considered to be non-resectional procedures (Table 2).

A valid correlation was established between the minor surgical procedure and osteitis (*p* = 0.001, OR = 0.216, 95% CI (0.080–0.577)), and regarding the significant surgical procedure, gangrene increased the risk for major amputation by more than two times (OR = 2.230, 95% CI (1.024–4.857)). Moreover, even if the number of infected Charcot foot diagnoses was limited (nine cases), the necessity of major amputation was increased in this subgroup (*p* = 0.008, OR = 5.316, 95% CI (1.354–20.877)).

Regarding the characteristics of patients at hospitalization, comorbidities such as anemia and diabetic renal disease were predictive of a major therapeutical approach, with *p* = 0.011, OR = 2.975, 95% CI (1.244–8.116) and *p* = 0.001, OR = 3.565, 95%CI (1.623–7.832), respectively.

One of the secondary objectives of this study was to identify the adverse or good outcomes of surgery, and it was demonstrated that significant amputation had only good outcomes (100%), *p* = 0.0031, OR = 0.842. 95% CI (0.796–0.892).

**Figure 4:**
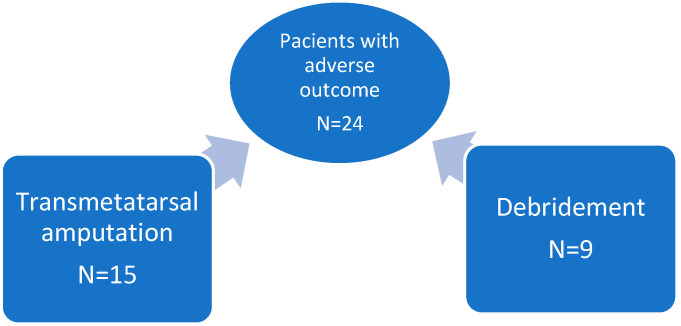
Type of surgical procedure in patients with adverse outcome.

As mentioned before, all the major amputations had good outcomes. Only several minor surgeries were interpreted as adverse surgical outcomes (*n* = 24) because they needed reintervention to restore the clean margins (nine cases) or minor amputation (15 patients) (Figure 4).

The characteristics of patients with adverse surgical outcomes were analyzed, which are detailed in Table 3. The main factors associated with negative outcomes are male gender, urban residence (there could be a bias regarding accessibility), long duration of diabetes mellitus, and insulin-dependency status. Still, without having statistical significance, all the *p* values identified were higher than 0.05 (*p* > 0.05). Furthermore, anemia at admission was borderline associated with adverse outcomes (*p* = 0.054).

Starting from the diagnostics of patients with adverse outcomes, it was demonstrated that osteitis was more frequent with this issue, in 11 cases of 35 (45.8%), without statistical validation compared with good outcomes.

Because most patients had infected lesions, it should be noted that in all cases, the treatment was performed according to the result of the antibiogram, and a study on the antibiotic treatment for diabetic foot infected lesions will be performed in the near future.

## 4. Discussion

Patients with diabetic foot lesions should always consult a specialized medical service because the prevention of lesions and their infection are essential factors that can reduce the number of debilitating surgeries by up to 50% [37,38].

**Figure 5:**
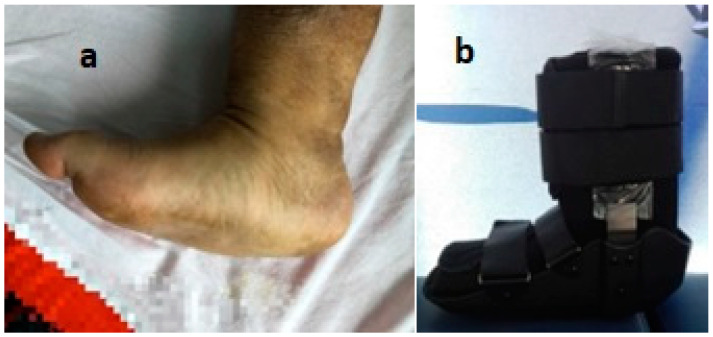
(**a**) Charcot foot. (**b**) Personalized neuropathic diabetic foot orthosis. Dr. I. Cantacuzino Clinical Hospital Collection.

It is mandatory for this kind of patient and their long-term prognosis to be treated by a multidisciplinary team (general surgeon, orthopedist, plastic surgeon, podiatrist). Even though adequate surgical management provides good postoperative results, as shown, special personalized footwear is mandatory to prevent a recurrence. The podiatrist’s role is essential to avoid lesions and recurrence after surgery, especially using off-loading therapy [37] (Figure 5).

The median duration of diabetes was calculated at 11.56 ± 6.5 years, a value below the publications in the literature, which attests to an average period of 25 years from the diagnosis of diabetes to the debut of peripheral neuropathic lesions [1].

The fact that most patients are male and with urban residence only confirms the data in the literature, which claim that due to the associated behaviors (alcohol consumption, tobacco) and unbalanced lifestyle significantly associated with urban life, the rate of complications related to diabetic foot pathology is higher [39,40].

Most patients admitted to our hospital had infected lesions (35.5%). The majority also had other complications of diabetes, such as nephropathy (17.1%), retinopathy (19.9%), and heart disease (63.3%); all of these complications represent risk factors for the appearance of diabetic foot lesions [41,42].

In the meta-analysis published in 2021 by Kaissar Yammine et al., the prevalence of renal diabetic impairment in patients with diabetic foot was 38.3%, a value twice as significant as the percentage reported in our study. Renal disease increases the risk of major amputation (*p* < 0.001), as Eggers demonstrated that the below-knee amputations and above-knee amputations are ten times higher in hemodialyzed patients versus no renal impairment. In our cohort, the association of nephropathy increased the risk of major amputation by more than three times, with *p* = 0.001, OR = 3.565, 95% CI (1.623–7.832) [43,44].

Costa et al. revealed that anemia is described to be the most predictive factor for major amputation (OR of 5.5, *p* < 0.0001). In our cohort, anemia was also found as a risk factor for major surgical procedures, with *p* = 0.011, OR = 2.975, 95% CI (1.244–8.116) [45].

**Figure 6:**
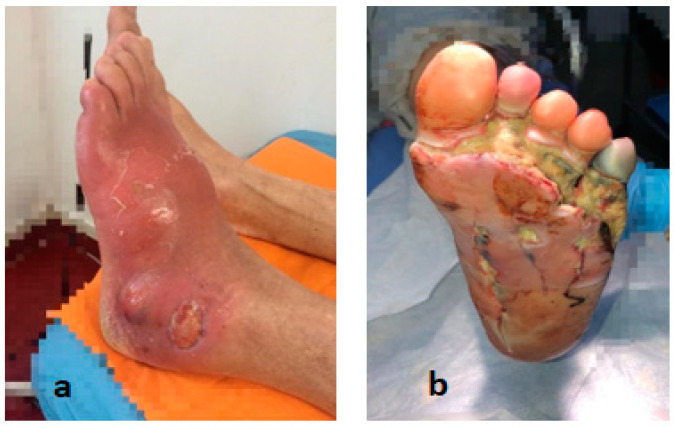
(**a**) Infected Charcot foot. (**b**) Foot gangrene. Dr. I. Cantacuzino Clinical Hospital Collection.

Regarding the surgical intervention, most of the minor surgical procedures were made for osteitis lesions in 92 patients (43.6%), then for infected ulcers (*n* = 61) (Figure 6). In most cases, major surgical procedures were chosen for advanced gangrene lesions (*n* = 12). The number of significant amputations is lower than the literature by establishing the proper surgical indications [46].

The follow-up after surgery in our study group concluded that a limited number of cases were considered adverse outcomes (needing reintervention), this issue being related to the diagnosis of osteitis and the anemia on admission status, both with no statistical significance (*p* > 0.05). A study conducted in the USA in 2017 highlighted that the need for an additional procedure was demanded in 56% of cases, especially regarding patients with minor surgical procedures [47].

The strength of our findings is that the study offers an objective analysis of the surgical management for patients with diabetic neuropathic foot based on the experience of a center specialized in this kind of pathology. Furthermore, a large number of patients and the surgery outcome shows that the surgical intervention made according to the type of lesion is the most relevant factor in helping the patients preserve their pelvic limb [48]. The limitation of this study is that it highlights the surgical results only from a tertiary center. The statistics are in agreement with the literature data, but further research will demonstrate if they can or cannot be extended for the entire surgical scientific society.

## 5. Conclusions

Romania’s limited accessibility in the medical–surgical service care of diabetic patients was a public health issue even before the COVID-19 pandemic. We are also raising the alarm about the lack of effective control of target glycemic values that leads to multiple complications of diabetes, including diabetic foot.

Osteitis is the most frequent diagnosis solved by minor amputation, but also the one that requires the most frequent surgical reintervention (adverse outcome).

The most important factors associated with major amputation (below or above the knee) were gangrene lesions and comorbidities such as anemia and diabetic kidney disease. However, surgery was mainly associated with a good outcome, preventing the increase in mortality or the risk of other systemic complications in this context.

This paper has shown that the focus of the surgical approach (minor and major amputations) to diabetic foot neuropathic complications is to decrease the number of cases with such complications. Currently, the data from the literature are poor in such cases since compliance with diabetes treatment recommendations is much higher in Western countries.

## Figures and Tables

**Table 1:** Characteristics of patients with osteitis and ulcer.

	All Subjects N = 251	Patients Diagnosed with Osteitis N = 97			Patients Diagnosed with Ulcer N = 69		
Characteristics	Value	Value	*p*-Value	OR (95% CI)	Value	*p*-Value	OR (95% CI)
Age at admission Mean ± SD ≤50 yo N (%) >50 and ≤70 yo N (%) >70 yo N (%)	61.21 ± 10.7 40 (15.9%) 160 (63.7%) 51 (20.3%)	61.08 ± 10.42	0.862	0.998 (0.975–1.022)	60.40 ± 11.44	0.884	0.994 (0.969–1.020)
Male sex N (%)	178 (70.9%)	60 (61.9%)	0.012 *	0.496 (0.284–0.861)	49 (71.1%)	0.983	1.007 (0.547–1.854)
Urban residents N (%)	148 (59.0%)	56 (57.7%)	0.753	0.920 (0.550–1.542)	36 (52.2%)	0.178	0.682 (0.390–1.192
Diabetes duration (years) Mean ± SD	11.56 ± 6.5	10.21 ± 5.49	0.015 *	0.949 (0.909–0.991)	10.83 ±5.72	0.419	0.978 (0.935–1.023)
Glucose level (mg/dL) Mean ± SD	200.83 ± 102.0	179.29 ± 92.29	0.007 *	0.997 (0.994–0.999)	185.98 ± 91.39	0.215	0.998 (0.995–1.001)
Insulin-dependent Status N (%)	113 (45.0%)	37 (38.1%)	0.082	1.580 (0.942–2.651)	30 (43.5%)	0.762	1.090 (0.624–1.905)
Retinopathy N (%)	50 (19.9%)	14 (14.4%)	0.084	0.553 (0.281–1.089)	16 (23.2%)	0.425	1.314 (0.671–2.573)
Nephropathy N (%)	43 (17.1%)	14 (14.4%)	0.368	0.727 (0.368–1.458)	10 (14.5%)	0.495	0.765 (0.355–1.651)
Cardiovascular diseases N (%)	159 (63.3%)	62 (63.9%)	0.882	1.041 (0.614–1.765)	39 (56.5%)	0.167	0.672 (0.381–1.183)
Anemia N (%)	153 (61.0%)	47 (48.5%)	0.001 *	0.426 (0.252–0.719)	38 (55.1%)	0.239	0.714 (0407–1.253)
Leukocytosis N (%)	89 (35.5%)	26 (26.8%)	0.023 *	0.529 (0.304–0.919)	24 (34.8%)	0.890	0.960 (0.537–1.716)

Abbreviations: N = number, % = percentage, SD = standard deviation, yo = years, * statistically significant result *p* < 0.05.

**Table 2:** Comparison of minor and major surgical procedures.

	Minor Surgical Procedure N = 211 (85.8%)	Major Surgical Procedure N= 35 (14.2%)	*p* Value	OR (95% CI)
**Diagnosis**				
Gangrene	40 (19.0%)	12 (34.3%)	0.040 *	2.230 (1.024–4.857)
Osteitis	92 (43.6%)	5 (14.3%)	0.001 *	0.216 (0.080–0.577)
Ulcer	61 (28.9%)	8 (22.9%)	0.460	0.729 (0.314–1.693)
Infected Charcot foot	5 (2.4%)	4 (11.4%)	0.008 *	5.316 (1.354–20.877)
Non-healing wound	6 (2.8%)	3 (8.6%)	0.095	3.203 (0.763–13.453)
Necrosis (dry)	5 (2.4%)	3 (8.6%)	0.055	3.863 (0.880–16.951)
Granulated wound	2 (0.9%)	0	0.563	0.857 (0.814–0.902)
**Patient’s characteristics**				
Insulin-dependent Status N (%)	95 (45.0%)	16 (45.7%)	0.939	0.973(0.474–1.994)
Male sex N (%)	148 (79.1%)	26 (74.3%)	0.618	1.230 (0.545–2.774)
Urban residents N (%)	124 (58.8%)	20 (57.1%)	0.857	0.935 (0.454–1.928)
Good outcome N (%)	187 (88.6%)	35 (100.0%)	0.031 *	0.842 (0.796–0.892)
Diabetes duration (yo) mean ± SD	11.23 ± 6.4	12.77 ± 6.4	0.118	1.036 (0.983–1.092)
Glucose level (mg/dL) mean ± SD	200.04 ± 101.1	208.70 ± 105.2	0.641	1.001 (0.997–1.004)
Anemia N (%)	121 (57.3%)	28 (80.0%)	0.011 *	2.975 (1.244–7.116)
Leukocytosis N (%)	74 (35.1%)	15 (42.9%)	0.375	1.389 (0.671–2.872)
Cardiovascular diseases N (%)	130 (61.6%)	25 (71.4%)	0.265	1.558 (0.711–3.412)
Retinopathy N (%)	40 (19.0%)	8 (22.9%)	0.590	1.267 (0.536–2.996)
Nephropathy N (%)	30 (14.4%)	14 (37.1%)	0.001 *	3.565 (1.623–7.832)

Abbreviations: N = number, % = percentage, SD = standard deviation, yo = years, * statistically significant result *p* < 0.05.

**Table 3:** Comparison of good and adverse outcomes after surgery.

Characteristics	Good Outcome N = 227	Adverse Outcome N = 24	*p*-Value	OR (95% CI)
Age at admission Mean ± SD	61.24 ± 11.0	60.88 ± 7.7	0.927	1.007 (0.965–1.051)
Male sex N (%)	162 (71.4%)	16 (66.7%)	0.630	0.802 (0.328–1.966)
Urban residents N (%)	135 (59.4%)	13 (54.2%)	0.615	0.805 (0.346–1.876)
Diabetes duration (yo) Mean ± SD	11.76 ± 6.5	9.71 ± 6.5	0.092	0.941 (0.868–1.019)
Insulin-dependent status N (%)	102 (44.9%)	11 (45.8%)	0.933	0.965 (0.414–2.224)
Glucose level (mg/dL) Mean ± SD	200.91 ± 101.8	200.08 ± 105.6	0.967	1.000 (0.996–1.005)
Retinopathy N (%)	46 (20.3%)	4 (16.7%)	0.675	0.787 (0.256–2.415)
Nephropathy N (%)	41 (18.1%)	2 (8.3%)	0.229	0.412 (0.093–1.824)
Anemia on admission N (%)	134 (59.0%)	19 (79.2%)	0.054	2.637 (0.951–7.314)
Cardiovascular diseases N (%)	147 (64.8%)	12 (50.0%)	0.154	0.544 (0.234–1.267)
Admission leukocytosis N (%)	78 (34.4%)	11 (45.8%)	0.264	1.616 (0.692–3.776)
**Diagnosis**				
Gangrene N = 52	49 (21.6%)	3 (12.5%)	0.296	0.519 (0.149–1.812)
Osteitis N = 97	86 (37.9%)	11 (45.8%)	0.447	1.387 (0.595–3.235)
Ulcer N = 69	62 (27.3%)	7 (29.2%)	0.847	1.096 (0.434–2.770)
Infected Charcot foot N = 9	8 (3.5%)	1 (4.2%)	0.602	1.190 (0.142–9.943)
Non-healing wound N = 9	8 (3.5%)	1 (4.2%)	0.602	1.190 (0.142–9.943)
Necrosis (dry) N = 8	7 (3.1%)	1 (4.2%)	0.558	1.366 (0.161–11.601)
Granulated wound N = 7	7 (3.1%)	0	0.383	0.902 (0.865–0.940)

Abbreviations: N = number, % = percentage, SD = standard deviation, yo = years.

## Data Availability

Not applicable.
